# Knowledge of bisphosphonate-related osteonecrosis of the Jaws among Mexican dentists

**DOI:** 10.4317/medoral.21433

**Published:** 2016-12-06

**Authors:** Ilan Vinitzky-Brener, Norma-Guadalupe Ibáñez-Mancera, Ana-Martha Aguilar-Rojas, Ana-Pilar Álvarez-Jardón

**Affiliations:** 1 DDS, Specialist in oral and maxillofacial surgery. Master in Education. Professor of Universidad Tecnológica de México (UNITEC-México Campus Marina), Professor of Anáhuac University, México. Exclusive private practice in oral and maxillofacial surgery; 2 DDS, Specialist in Oral Medicine. Professor of Anáhuac University, México, and CICS-UST, IPN. Exclusive private practice in oral medicine; 3 DDS. Anáhuac University, México. First year resident, Orthodontics Department of Universidad Tecnológica de México, UNITEC; 4 Dental school resident. Anáhuac University, México Norte

## Abstract

**Background:**

Bisphosphonate-related osteonecrosis is an infrequent but potentially serious complication. Its treatment remains complex, and in some cases can be mutilating. Prevention, a correct diagnosis and opportune management are crucial.

**Material and Methods:**

A cross-sectional study was made, interviewing 410 dentists with the aim of assessing their knowledge of the subject.

**Results:**

Practically all of the dental professionals (99.7%) were found to lack sufficient knowledge of the prevention, diagnosis and management of bisphosphonate-related osteonecrosis.

**Conclusions:**

Actions including increased diffusion in the professional media and inclusion of the subject in training programs are needed in order to enhance the knowledge of bisphosphonate-related osteonecrosis among dentists and thus prevent complications in this group of patients.

**Key words:**Knowledge, mexico, osteonecrosis, bisphosphonates.

## Introduction

Bisphosphonates are drugs used to prevent and treat certain bone disorders, including osteoporosis / osteopenia, multiple myeloma, bone metastases associated to certain types of cancer, hypercalcemia, Paget’s disease, etc. (1). The first therapeutic bisphosphonate was etidronate, introduced on the market in 1960 for the management of Paget’s disease. The mechanism of action of these drugs involves the induction of osteoclast apoptosis, resulting in the inhibition of bone reabsorption. Many agents belonging to this drug group are currently available in Mexico, and they are widely used in different medical specialties (2,3). In 2003, Marx published the first report associating bisphosphonate use to the exposure of maxillary bone, one year later Ruggiero published a series of 63 patients with bisphosphonate-related osteonecrosis of the jaws (BRONJ) (4). Since then, disorders of this kind have been widely reported in the medical and dental literature.

On entering the key word “bisphosphonate-related osteonecrosis”, specialized scientific search tools such as PubMed currently yield over 960 articles referred to this topic. Even non-specialized popular websites such as Wikipedia mention the risk of osteonecrosis of the jaws associated to the use of bisphosphonates. Bisphosphonate-related osteonecrosis is an infrequent but potentially serious problem (1,4). Its treatment remains complex, and in some cases can be aggressive and mutilating (5,6). Despite the extensive scientific and non-scientific literature on the subject, many Mexican physicians and dentists lack the knowledge about bisphosphonate-related osteonecrosis, or underestimate its risks - a fact that can have important consequences for patient health.

The present study was carried out to evaluate the knowledge of bisphosphonate-related osteonecrosis among Mexican dentists, including its risk factors and management options, with a view to defining proper strategies capable of reducing the frequency of this complication.

## Material and Methods

A cross-sectional study was made, interviewing a probabilistic sample of 410 dentists with the aim of assessing their knowledge of the subject. The dental professionals came from both private and public general or specialized practice in 25 states of the country. The questionnaire was divided into areas. A first area addressed general information on the dental professionals: training, specialization (if any), type of practice, years of professional experience, participation in updating training courses, the Mexican state in which professional practice is carried out, membership in associations or specialized boards, and teaching activities. A second area wash focused to assess the degree of knowledge about bisphosphonate-related osteonecrosis, the questionnaire asked about the therapeutic indications of bisphosphonates, the drug substances involved, brand names, risk factors, the diagnostic criteria and treatment options. Lastly, a third area was used to asses a clinical case involving a patient receiving bisphosphonate treatment and requiring dental extraction. The answers obtained were evaluated by experts, based on the most recent literature on the subject. The study was reviewed and approved by the institutional ethics committee and written consent was obtained for all the participants in the study.

## Results

A total of 410 interviews were carried out among dentists from 25 states throughout the country – the predominant settings being Mexico City (n = 134; 32.7%) and Mexico state (n = 148; 36.1%). A total of 249 dentists (60.7%) were dedicated to general practice, 122 (29.8%) were specialists, 36 (8.8%) had training at master degree level, and three (0.7%) held a PhD. The distribution by specialties was as follows: orthodontics 67 (16.3%), prosthodontics and oral rehabilitation 22 (5.4%), pediatric dentistry 18 (4.4%), endodontics 14 (3.4%), maxillofacial surgery 13 (3.2%), periodontics 9 (2.2%) oral medicine 4 (1%) and others 10 (2.4%). Of those interviewed 71% were dedicated to private practice, 15% to public practice, and 13.4% to both. Only 34% reported being affiliated to some association or specialized board. With regard to teaching activities, 115 of the professionals (28%) were or had been involved in academic activities. In turn, 215 of the dentists (52%) reported having attended between 1-5 congresses during the last 5 years.

The findings referred to general knowledge of bisphosphonates and bisphosphonate-related osteonecrosis are shown in  Table 1.

**Table 1:**
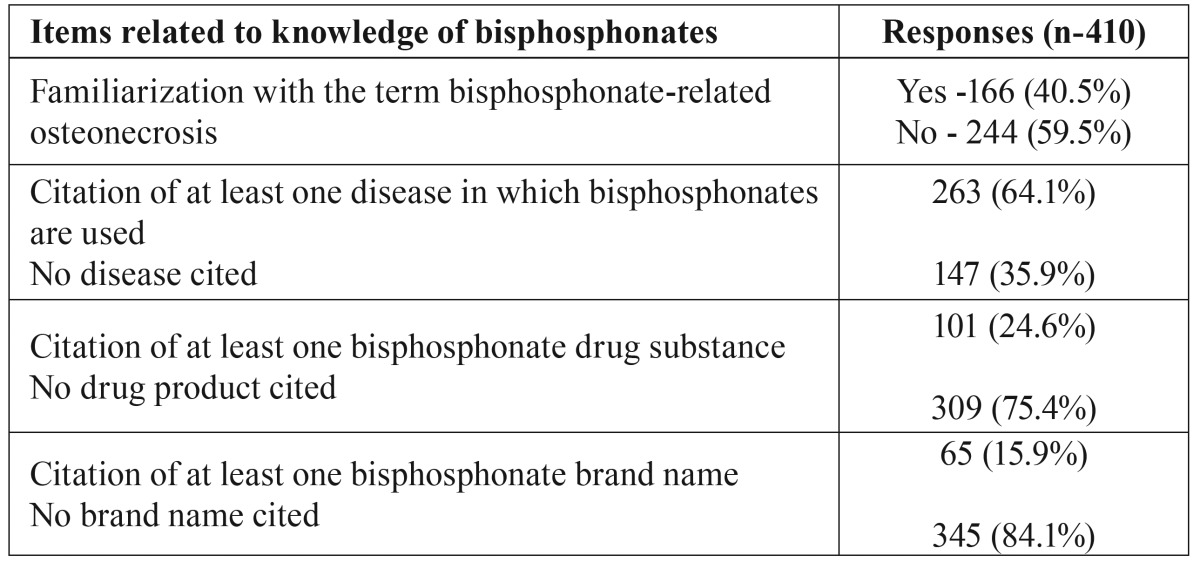
General questionnaire items referred to bisphosphonates and bisphosphonate-related osteonecrosis.

The findings referred to knowledge of diagnostic criteria, risk factors and treatment options in bisphosphonate-related osteonecrosis are shown in  Table 2.

**Table 2:**
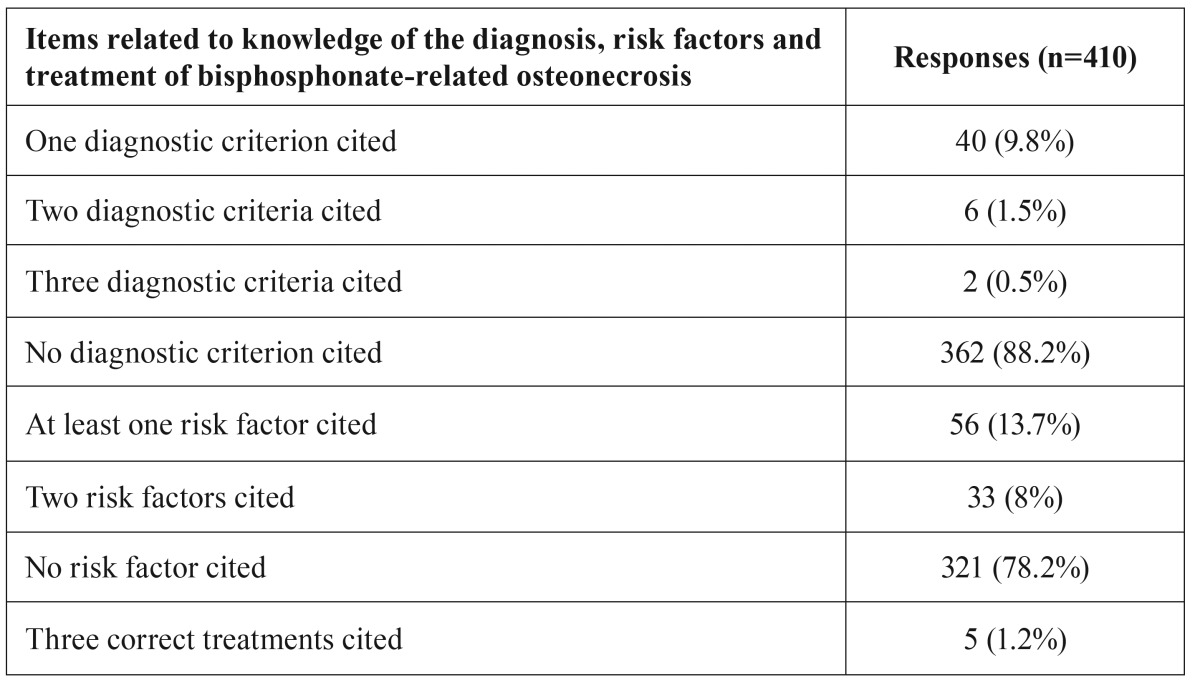
Level of knowledge among the dentists referred to diagnostic criteria, risk factors and treatment options in bisphosphonate-related osteonecrosis.

Regarding assessment of the clinical case presented in the questionnaire, 140 of the dentists (32.2%) stated that they would perform extraction (90 with prior antibiotic administration and 50 without), 92 (22.4%) would not perform extraction, and 166 (40.5%) would refer the patient to specialized care. These data were statistically significant (*p*=0.01).

Specific criteria applied to the answers were established to determine how many of those interviewed had sufficient knowledge of the subject and could adequately treat a patient receiving or having received bisphosphonates. In this regard, 407 (99.3%) of the professionals were found to lack sufficient knowledge of the prevention, diagnosis and management of bisphosphonate-related osteonecrosis, and only three (0.7%) met the established criteria. These three professionals were specialized in maxillofacial surgery, oral medicine and periodontics, respectively.

## Discussion

The results obtained in our study are a reason for concern, since they indicate that practically all of the dental professionals (99.7%) lacked sufficient knowledge for the adequate diagnosis and management of patients receiving bisphosphonates and who require dental care.

In similar studies conducted in other countries, Yoo *et al.* in Korea found 56.5% of a series of 254 dentists to be familiarized with bisphosphonate-related osteonecrosis, and 31.4% recorded patient antecedents referred to these drugs in the clinical history. The authors found that most of those interviewed were not aware of the guidelines of the American Association of Oral and Maxillofacial Surgeons (7). In Ontario (Canada), Alhussain *et al.* found 60% of the dentists interviewed to have adequate knowledge of bisphosphonate-related osteonecrosis. On the other hand, 50% of the professionals did not feel comfortable treating patients using bisphosphonates, and 23% claimed to follow the published guidelines referred to surgical treatment (8).

An interesting observation in our study is the fact that although 166 (40.5%) of those interviewed claimed to be familiarized with the term “bisphosphonate-related osteonecrosis”, and 144 (34%) reported having read some article or attended some conference on the subject, only 3 (0.7%) of the dental professionals actually demonstrated sufficient knowledge in this field.

Of the global participants in the study, 140 (34.1%) reported that they would perform tooth extraction in a patient receiving bisphosphonates - a situation that would pose a risk of developing osteonecrosis. Considering that drugs of this kind are widely used in the general population, these findings indicate that a relevant proportion of individuals subjected to oral surgical procedures would be at risk of developing osteonecrosis. This situation may not have serious consequences if correct diagnosis is established and adequate treatment is provided; however, if the diagnosis is delayed or further surgical procedures are carried out, the consequences for the patient may prove to be serious - with important expenditure in terms of healthcare costs and resources.

Of the professionals who answered that they would perform extraction, 80 (57%) were general dentists and 22 (15.7%) were orthodontists. This suggests that dentists lacking postgraduate training have even less knowledge in this area.

It is crucial to analyze why we have such poor results in this field in our country, in order to adopt corrective measures and avoid complications in patients at risk. An important aspect is the lack of specialization and postgraduate training. The dental professional census in Mexico corresponding to 2010 reflected the existence of 151,622 dentists, of which only 6.5% had specialized training, and 0.75% and 0.035% held a master degree and PhD, respectively (9). The percentage of dentists with postgraduate training is clearly very low. Another important factor is the lack of attending to updating courses. In effect, we found that 59% of those interviewed attended less than one congress a year during the last 5 years, and 5% claimed that did not attended any such congresses at all. Lastly, another significant element to be considered would be the number of scientific articles read by the dental professionals, though we lacked data to explore this factor.

## Conclusions

The results of our study reveal very limited knowledge of bisphosphonate-related osteonecrosis among Mexican dentists. In effect, a full 99.3% of those interviewed did not have sufficient knowledge regarding drugs, diagnosis and treatment in this field - a fact that could pose an important problem for patients at risk. In our opinion, the situation could be improved through increased diffusion of the subject at congresses and scientific events; communications issued by the health institutions or dental associations; and the inclusion of bisphosphonate-related osteonecrosis in both pre- and postgraduate training programs. We also consider it advisable for dental professionals to document bisphosphonate use in the clinical history - questioning their patients directly in this regard.

On the basis of the results obtained, we consider it important to conduct similar studies targeted to general and specialized physicians who prescribe these drugs, in order to evaluate their knowledge in the field and know whether or not they adequately warn their patients on the oral cavity risks involved.
